# Association of polygenic risk for bipolar disorder with grey matter structure and white matter integrity in youth

**DOI:** 10.1038/s41398-023-02607-y

**Published:** 2023-10-18

**Authors:** Xinyue Jiang, Clement C. Zai, Kody G. Kennedy, Yi Zou, Yuliya S. Nikolova, Daniel Felsky, L. Trevor Young, Bradley J. MacIntosh, Benjamin I. Goldstein

**Affiliations:** 1 Centre for Youth Bipolar Disorder, Centre for Addiction and Mental Health, Toronto, ON Canada; 2 Department of Pharmacology & Toxicology, University of Toronto, Toronto, ON Canada; 3 Centre for Addiction and Mental Health, Toronto, ON Canada; 4 Department of Psychiatry, University of Toronto, Toronto, ON Canada; 5 Division of Biostatistics, Dalla Lana School of Public Health, University of Toronto, Toronto, ON Canada; 6 Sandra E Black Centre for Brain Resilience and Recovery, Sunnybrook Research Institute, Toronto, ON Canada; 7 Department of Medical Biophysics, University of Toronto, Toronto, ON Canada; 8 Hurvitz Brain Sciences Program, Sunnybrook Research Institute, Toronto, ON Canada

**Keywords:** Clinical genetics, Bipolar disorder, Clinical genetics

## Abstract

There is a gap in knowledge regarding the polygenic underpinnings of brain anomalies observed in youth bipolar disorder (BD). This study examined the association of a polygenic risk score for BD (BD-PRS) with grey matter structure and white matter integrity in youth with and without BD. 113 participants were included in the analyses, including 78 participants with both T1-weighted and diffusion-weighted MRI images, 32 participants with T1-weighted images only, and 3 participants with diffusion-weighted images only. BD-PRS was calculated using PRS-CS-auto and was based on independent adult genome-wide summary statistics. Vertex- and voxel-wise analyses examined the associations of BD-PRS with grey matter metrics (cortical volume [CV], cortical surface area [CSA], cortical thickness [CTh]) and fractional anisotropy [FA] in the combined sample, and separately in BD and HC. In the combined sample of participants with T1-weighted images (*n* = 110, 66 BD, 44 HC), higher BD-PRS was associated with smaller grey matter metrics in frontal and temporal regions. In within-group analyses, higher BD-PRS was associated with lower CTh of frontal, temporal, and fusiform gyrus in BD, and with lower CV and CSA of superior frontal gyrus in HC. In the combined sample of participants with diffusion-weighted images (*n* = 81, 49 BD, 32 HC), higher BD-PRS was associated with lower FA in widespread white matter regions. In summary, BD-PRS calculated based on adult genetic data was negatively associated with grey matter structure and FA in youth in regions implicated in BD, which may suggest neuroimaging markers of vulnerability to BD. Future longitudinal studies are needed to examine whether BD-PRS predicts neurodevelopmental changes in BD vs. HC and its interaction with course of illness and long-term medication use.

## Introduction

Bipolar disorder (BD) is a severe recurrent mood disorder characterized by episodes of mania and/or hypomania and depression, affecting 2–3% of youth worldwide [1, 2]. BD is highly heritable and polygenic, with a heritability of 60–85% [3, 4]. Polygenic risk score(s) (PRS), often calculated as the weighted sum of single-nucleotide polymorphism (SNP) risk alleles identified by genome-wide association studies (GWAS), can be studied as an index of an individual’s genetic liability for BD [5]. Previous work in adults has shown that PRS for BD (BD-PRS) can significantly differentiate BD cases, unaffected relatives, and controls [6–11], and are associated with a greater burden of mania/hypomania and diagnostic conversion from major depressive disorder to BD [12–16]. There is also replicated evidence of higher BD-PRS among youth with first-degree family history of BD [9, 17, 18]. Recent findings from a prospective study showed that BD-PRS was elevated in parents with BD and their offspring, particularly in offspring who developed BD and whose parents had early onset BD [17, 18].

In addition to BD-specific genetic factors, there is robust evidence of brain anomalies in both adults and youth with BD, including grey matter reduction in frontal, temporal, and limbic regions [17–23], as well as widespread white matter microstructural abnormalities, as indicated by reduced fractional anisotropy (FA) across major white matter tracts identified in diffusion tensor imaging (DTI) studies [24–31]. Incorporating PRS in neuroimaging studies has the potential to inform whether the observed BD-related neuroimaging characteristics can be attributed to genetic factors [32, 33]. In contrast to individual SNPs, the PRS approach reflects a greater proportion of the shared genetic factors for BD, and related findings may therefore be more generalizable to all individuals with BD. In both general adult population and adults with or at risk for BD, BD-PRS have been associated with differences in grey matter volume and thickness in various regions relevant to BD, including frontal and cingulate regions [33–37], although negative findings have also been reported [38–40]. In a study of adults at familial risk for BD and a study of healthy adults, no significant associations were identified between BD-PRS and FA [39, 41]. However, no studies have examined such associations within a BD sample as yet.

Studying neuroimaging genetics in youth provides an opportunity to identify core neurobiological characteristics of the illness in a population with less impact of repeated affective episodes and other confounding factors associated with illness course, environmental factors, and medications as compared to adults [21]. Thus far only three studies have examined associations of BD-PRS with grey matter volume and white matter integrity in the general pediatric population [42–44]. However, this topic has not been addressed in youth with BD.

To address related gaps in knowledge, this study aims to examine whether grey matter structure and white matter integrity are associated with BD-PRS in youth. Our primary hypothesis was that higher BD-PRS would be associated with smaller grey matter metrics (cortical volume [CV], cortical surface area [CSA], and cortical thickness [CTh]) and lower FA in the overall sample. Secondary analyses further examined the associations separately in the BD group and healthy controls (HC), to explore whether the pattern of findings in the overall sample would be evident in each diagnostic group. Additionally, as medication use has been shown to affect brain imaging metrics [18, 22, 45–47], exploratory analyses further investigated the association of current and lifetime use of lithium, second-generation antipsychotics (SGA), and lamotrigine, as well as their interaction with BD-PRS, with significant clusters identified in the BD group. By employing a multimodal whole-brain approach, the goal of this preliminary study is to generate findings that can be compared to prior adult findings, and that can serve to guide future youth BD studies on this topic.

## Methods

### Participants

78 participants underwent both T1-weighted and diffusion-weighted MRI sequences. In addition, 32 participants underwent T1-weighted MRI sequence but did not participate in the DTI study, and 3 participants only had diffusion-weighted MRI images because their T1-weighted MRI images were excluded from analyses due to poor image quality (see Image Acquisition and Processing). In total, 110 participants with T1-weighted MRI (*n* = 66 BD, *n* = 44 HC) and 81 participants with diffusion-weighted MRI data collection (*n* = 49 BD, *n* = 32 HC) were included in the analyses.

Participants were between the ages of 13–20 years and were genetically European. BD participants (type I, II, or not otherwise specified [NOS]) were recruited through a subspecialty clinic at an academic health science center in Toronto, Ontario Canada. HC were recruited from the community through advertisements. Exclusion criteria included: known existing cardiac conditions; autoimmune or inflammatory conditions; taking anti-inflammatory, antiplatelet, antilipidemic, antihypertensive, or hypoglycemic agents; infectious illness in the 14 days before the study; any MRI contraindications (i.e. any metal in the body, claustrophobia, etc.); any severe neurological or cognitive impairments, or unable to provide informed consent. Additionally, HC participants had no major or recent psychiatric disorders (no lifetime mood or psychotic disorders, no recent alcohol or drug dependence in the past 3 months, and no recent anxiety disorders within the past 3 months) and no family history of BD or psychotic disorder (first and second degree relatives).

Written informed consent was obtained from all participants, as well as their parent(s) or guardian(s). Ethical approval was granted by Sunnybrook Research Institute Research Ethics Board. All data was collected at Sunnybrook Research Institute, and was transferred with the Centre for Youth Bipolar Disorder’s relocation to the Centre for Addiction and Mental Health (CAMH). Ethical approval was also granted by CAMH Research Ethics Board.

### Diagnostic interview and symptom ratings

BD diagnoses and other clinical information were obtained via interview with youth and parent(s) using the Kiddie-Schedule for Affective Disorders and Schizophrenia for School-Age Children, Present and Lifetime version (K-SADS-PL) [48]. The K-SADS-PL is a semi-structured interview with both parent and youth that is used to determine present and lifetime history of psychiatric illness in children and adolescents between the ages of 7 and 18 years, according to the Diagnostic and Statistical Manual of Mental Disorders, Fourth Edition (DSM-IV) (APA, 2000) criteria. Diagnoses in this study were based on the DSM-IV criteria, as participants were enrolled from 2014 to 2019 and the DMS-5 version of the K-SADS-PL was not available until 2016. BD subtypes I and II were defined using the DSM-IV criteria. Diagnosis of BD-NOS was based on operationalized criteria from the Course and Outcome of Bipolar Illness in Youth (COBY) study for the duration of symptoms (minimum 4 h/day) and number of hypomanic days (minimum 4 in lifetime), while retaining DSM 5 symptom count requirements (i.e. 3 symptoms when elation was the primary symptom, 4 symptoms when irritability was the primary symptom) [49]. Lifetime use of lithium and SGA was ascertained via the K-SADS-PL and was computed as a “yes” or “no” variable. Details regarding clinical measures are described in the Supplementary Materials and Methods.

All interviews were performed by trained study personnel with either Bachelor’s or Master’s degree in a health-related field and completed comprehensive K-SADS-PL training under the supervision of the senior author (B.I.G.), a licensed child and adolescent psychiatrist. All diagnostic and symptom ratings were reviewed and confirmed by a licensed child and adolescent psychiatrist.

### Polygenic risk score

A saliva sample (~2 mL) was collected from each participants in an Oragene OG-500 DNA kit (DNA Genotek Oragene-500 kits; DNA Genotek Inc., Ottawa, Canada). Participants were instructed to abstain from eating, drinking, smoking, and chewing gum 30 min prior to saliva collection. Detailed methods regarding DNA extraction, genotyping, genetic quality control, and imputation can be found in Supplementary Materials and Methods.

BD-PRS was derived from GWAS summary statistics from the latest Psychiatric Genomics Consortium study on BD in adults of European ancestry, in which 64 genome-wide significant genomic loci were identified [50]. BD-PRS was calculated using PRS-CS-auto, a Bayesian-based method that places a continuous shrinkage prior to the effect sizes of SNPs in the discovery GWAS summary statistics [51]. PRS-CS-auto was used because it demonstrated better predictive accuracy across a wide range of genetic architectures with computational scalability, and does not require a tuning dataset [52]. Moreover, PRS-CS-auto has been used in other studies of bipolar disorder and suicide attempts [53–55]. The BD GWAS summary statistics data were processed using the PRS-CS software, with default settings, automatic estimation of the global shrinkage parameter (PRS-CS-auto), and the European subset of the 1000 Genomes Project Phase 3 dataset as the LD reference panel. PLINK 1.9 was used to sum all the effect alleles of 944420 SNPs, weighted by the effect sizes derived from PRS-CS-auto, into PRS for each individual in our target cohort. The derived PRS were subsequently standardized to a mean of 0 and an SD of 1.

### Image acquisition and processing

#### T1-weighted imaging

T1-weighted images were collected using a 3 Tesla (3 T) Phillips Achieva system with an 8-channel head receiver coil and body coil transmission (Philips Medical Systems, Best, Netherlands). The acquisition parameters were as follow: repetition time (TR) 9.5 ms, echo time (TE) 2.3 ms, inversion time (TI) 1400 ms, spatial resolution 0.94 × 1.17 × 1.2 mm (nearly 1 mm isotropic), 256 × 164 × 140 matrix, flip angle 8°, scan duration 8 min 56 s.

Image processing of the T1-weighted images was performed using FreeSurfer (V6.0) software. (http://surfer.nmr.mgh.harvard.edu.myaccess.library.utoronto.ca/) [56]. This includes removing non-brain tissue via automated skull stripping [57], Talairach transformations, parcellation of white and grey matter [58], intensity normalization [59], tessellation of the grey matter white matter boundary [60], and topology correction [61]. The brain was then inflated to enable registration to a spherical atlas which is based on individual cortical folding patterns to match cortical geometry across subjects [58]. The registered brain was then mapped to the Desikan-Killiany probabilistic atlas for cortical parcellation [62]. To facilitate the vertex-wise analysis, surface-based smoothing with a full-width at half-maximum of 15 mm was employed before mapping CV, CSA, and CTh data to the canonical template. Prior to pre-processing, T1-weighted images were visually inspected by 3 independent raters to assess the quality of images (eg. artifacts, contrast between white matter and grey matter, or otherwise poor image quality) and the accuracy of parcellation (e.g., correctly labeled structures). For each image, a score between 0 and 3 was given based on overall image quality. If the scores between raters were incongruent, images were inspected a second time to ensure that a consensus was achieved following discussion. Images with a score of 3 (poor quality) were excluded from pre-processing and analyses. Images with poor parcellation accuracy were manually edited. Three participants (1 BD and 2 HC) were excluded from analyses due to poor image quality. 12 images were edited following QC (6 BD and 6 HC).

#### Diffusion tensor imaging

Diffusion-weighted data were acquired using a single-shot, spin-echo planar imaging (EPI) sequence on a 3 Tesla Philips Achieva MRI scanner (Philips Medical Systems, Best, Netherlands). Diffusion data were collected along 32 gradient directions at a *b* value of 1000 s/mm^2^ for each participants. Seven images with no diffusion weighting were obtained. The acquisition parameters were as follow: TR/TE = 9150/55 ms; flip angle=90; field-of-view [FOV] = 224 × 224; fifty-two 3mm-thick slices; matrix size = 128 × 128; acquisition duration: 6 min 27 s. The axial imaging plane was prescribed obliquely to align with the anterior-to-posterior commissure.

FMRIB Software Library (FSL) tools were used to perform diffusion data processing and analysis (FMRIB, Oxford Center for Functional MRI of the Brain, University of Oxford) [63]. Diffusion-weighted images were eddy-current corrected and brain extracted. This is followed by tensor fitting using DTIFIT to calculate DTI metrics. Individual FA maps were computed from the tensor eigenvalues (λ1, λ2, λ3). Individual participants’ major WM tracts were aligned with a mean FA skeleton using Tract-based spatial statistics (TBSS) for voxel-wise statistics. Specifically, all participants’ FA maps were registered to a study-specific target chosen from the youth sample. The registered maps were then transformed into MNI space. Next, a skeleton of the mean of all FA maps in standard space was calculated, and a threshold of 0.3 was applied to exclude non-WM voxels and to remove the high inter-subject variability at tract extremities [30, 64]. Two independent raters inspected the quality after each step.

### Statistical analysis

Demographic and clinical group differences were evaluated using SPSS Version 27. Shapiro-Wilks test and Levene’s test were used to check normality and equal variance assumptions of all continuous variables. Group differences were evaluated using *t*-tests for continuous variables and chi-squared tests for categorical variables. Mann-Whitney *U*-tests were used for variables that were not normally distributed. Statistical significance was set at two-sided *p* < 0.05

General linear models (GLMs) were used to examine the main effects of BD-PRS on each grey matter metric (CV, CSA, and CTh) and DTI metric (FA). Primary analyses were conducted in the combined sample of BD and HC to maximize power, and because we anticipated the direction of any association would be similar in both diagnostic groups. Secondary analyses further examined the associations within the BD group and HC group separately. Age and sex were controlled as covariates. Aligned with the ENIGMA protocol [18], intracranial volume (ICV) was included as covariate when examining CV and CSA. ICV was not included as a covariate in the model for CTh analyses as it is not correlated with CTh (see Table S2 in Supplementary Materials and Methods) [65]. In addition, given the small sample size, primary analyses covaried for the top two genetic principal components selected based on the Scree plot (explaining 40% of variance). Genetic principal components are derived from principal component analysis of genetic data, and are controlled for in genetic analyses to account for population stratification (i.e. the difference in allele frequencies between subpopulations in a study due to ancestry difference) [66].

For grey matter metrics, vertex-wise analyses were performed using Freesurfer. Results were thresholded at *p* < 0.05, and were corrected for multiple comparisons using permutation testing within the FreeSurfer package (10,000 permutations). Cluster-wide p values were then calculated as the probability of detecting a cluster of that size by chance and reported for each significant cluster. Corresponding grey matter regions were identified using the Desikan-Killiany atlas. For DTI metrics, a group skeletonized 4D FA image was generated and used as the input for voxel-wise analysis using the FSL randomize tool [67]. The significance threshold was set at *p* < 0.05 and results were corrected for multiple comparisons using the family-wise error rate correction with the “Threshold-Free Cluster Enhancement” thresholding option with 5000 permutations [68]. The FSL Cluster tool was used to obtain cluster size, anatomical coordinates, and peak p values of significant clusters. Corresponding white matter tracts were identified using the CBM-DTI-81 white-matter labels. For visualization purposes, significant FA clusters were thickened using *tbss_fill* in FSL. Significant clusters identified from the vertex- and voxel-wise analyses were used as masks to extract grey and white matter metric values for each participant. The β values for the association of BD-PRS with grey and white matter metrics for the significant clusters were then calculated in SPSS using the aforementioned GLMs. Due to the paucity of literature investigating the associations of BD-PRS with neuroimaging modalities in youth BD populations, and the preliminary nature of this study, we present results that were corrected for multiple comparisons for whole-brain vertex/voxel-wise analyses within each imaging modality (i.e. CV, CSA, CTh, and FA) using permutation tests described above, but were uncorrected across the different modalities, in order to inform future studies.

Exploratory analyses examined the main effect of current and lifetime use of lithium, SGA, and lamotrigine, as well as their interaction with BD-PRS on significant clusters identified in the BD group. Additionally, to parse the potential effect of sex, exploratory analyses also examined the main effect of sex and its interaction with BD-PRS on significant clusters identified from primary analyses.

## Results

### Demographic and clinical characteristics

Demographic characteristics of all participants are presented in Table 1. Compared to the HC group, the BD group was significantly older, with higher Tanner stage, and had a greater proportion of female participants. For participants with T1-weighted images, BMI was significantly higher in BD than HC. Clinical characteristics of the BD group are summarized in Table S1 in Supplementary Materials and Methods. BD-PRS was significantly higher in the BD group than the HC group (OR = 1.66, *p* = 0.03, 95%CI = 1.04–2.64).

**Table 1:** Demographic characteristics of study participants.

	With T1-weighted images (*n* = 110)					With DTI images (*n* = 81)				
	BD (*n* = 66)	HC (*n* = 44)	Test statistic	*p*-value	Effect size	BD (*n* = 49)	HC (*n* = 32)	Test statistic	*p*-value	Effect size
Age, years	17.3 ± 1.5	16.5 ± 1.6	*t* = 2.71	**0.01**	*d* = 0.53	17.9 ± 1.8	16.9 ± 1.8	*t* = 2.26	**0.03**	*d* = 0.51
Sex (n, % female)	45 (68.2)	21 (47.7)	χ^2^ = 4.60	**0.03**	*V* = 0.21	34 (69.4)	14 (43.8)	χ^2^ = 5.27	**0.02**	*V* = 0.26
SES	4.2 ± 0.9	4.4 ± 1.0	*U* = 1270.50	0.22	*d* = 0.16	4.3 ± 1.0	4.4 ± 1.0	*U* = 727.50	0.54	*d* = 0.10
Intact family (n, %)	39 (60.0)	31 (70.5)	χ2 = 1.25	0.26	*V* = 0.11	31 (64.6)	21 (65.6)	χ^2^ = 0.01	0.92	*V* = 0.01
Tanner Stage (1–5)	4.5 ± 0.7	4.2 ± 0.6	*U* = 1756.50	**0.04**	*d* = 0.37	4.6 ± 0.6	4.1 ± 0.7	*U* = 1066.0	**<0.001**	*d* = 0.82
BMI (adjusted)	23.9 ± 4.7	21.5 ± 2.9	*U* = 1923.0	**0.004**	*d* = 0.61	24.0 ± 5.0	22.1 ± 3.1	*U* = 917.0	0.12	*d* = 0.43

Values for all continuous variables are presented as mean ± standard deviation and categorical variables are presented as n (% within group). Effect size = Cohen’s d or Cramer’s V.

*BD* bipolar disorder, *HC* healthy controls, *SES* socio-economic status, *BMI* body mass index, adjusted for clothing differences (i.e. weight - 1.4 kg for long pants and long shirt/sweatshirt, −1.1 kg for short pants or short-sleeves, −0.9 kg for short pants and short-sleeves).

### PRS and grey matter structure

**Fig. 1:**
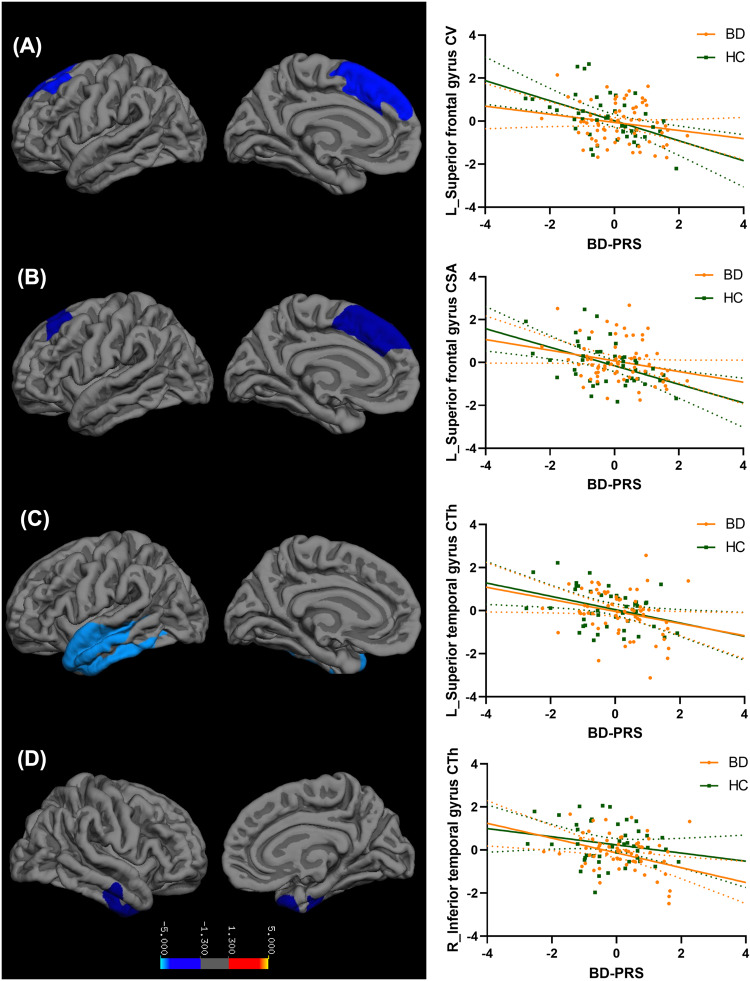
Association between BD-PRS and grey matter metrics of the significant clusters identified in the combined sample of BD and HC. Higher BD-PRS was associated with smaller (**A**) left superior frontal gyrus CV, (**B**) left superior frontal gyrus CSA, (**C**) left superior temporal gyrus CTh, and (**D**) right inferior temporal gyrus CTh. The *y*-axis is labeled by the main region of significant clusters, and the value on *y*-axis indicates the standardized residuals of grey matter metrics of the significant clusters, adjusted for age, sex, and two genetic principal components. CV and CSA were also adjusted for intracranial volume. The color bar represents the range log transformed *p*-values.

**Fig. 2:**
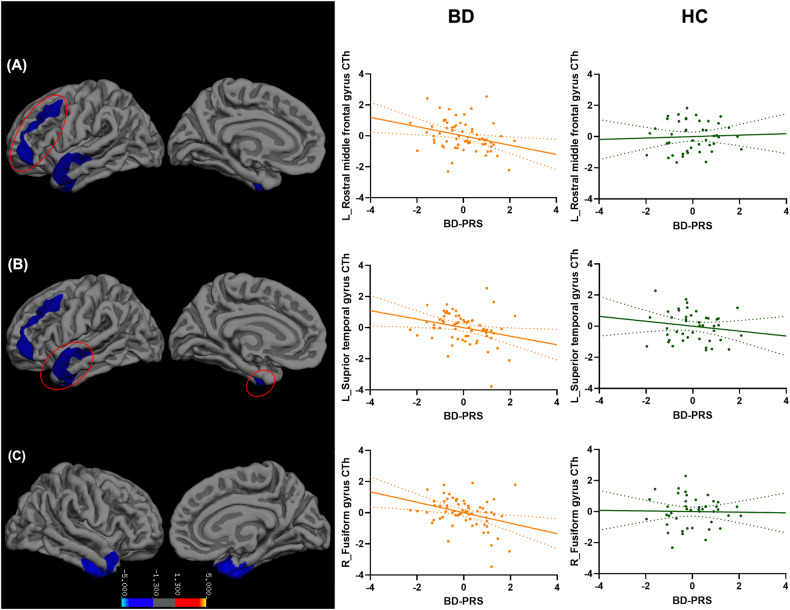
Association between BD-PRS and grey matter metrics of the significant clusters identified within BD. Higher BD-PRS was associated with thinner (**A**) left rostral middle frontal gyrus, (**B**) left superior temporal gyrus, and (**C**) right fusiform gyrus in the BD group (Orange). Similar associations were not observed in the HC group (Green). The *y*-axis is labeled by the main region of significant clusters, and the value on *y*-axis indicates the standardized residuals of grey matter metrics of the significant clusters, adjusted for age, sex, and two genetic principal components. The color bar represents the range log transformed *p*-values.

**Fig. 3:**
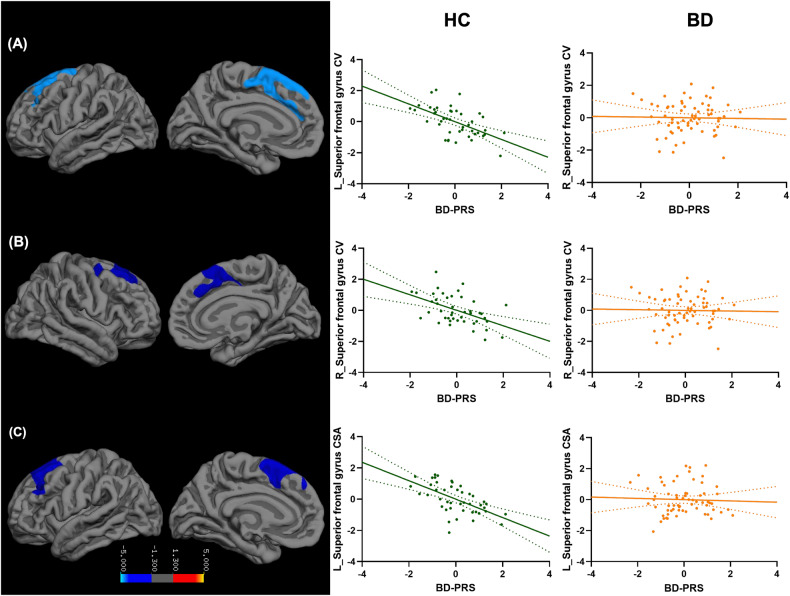
Association between BD-PRS and grey matter metrics of the significant clusters identified within HC. Higher BD-PRS was associated with smaller (**A**) left superior frontal gyrus CV, (**B**) right superior frontal gyrus CV, and (**C**) left superior frontal gyrus CSA in the HC group (Green). Similar associations were not observed in the BD group (Orange). The *y*-axis is labeled by the main region of significant clusters, and the value on *y*-axis indicates the standardized residuals of grey matter metrics of the significant clusters, adjusted for age, sex, and two genetic principal components. CV and CSA were also adjusted for intracranial volume. The color bar represents the range log transformed *p*-values.

The main effects of BD-PRS on grey matter structure in the combined sample of BD and HC, within BD, and within HC are presented in Table 2. In the combined sample (Fig. 1), higher BD-PRS was significantly associated with smaller CV and CSA of the left superior frontal gyrus (β = −0.25, *p* = 0.002; β = −0.23, *p* = 0.03; respectively). Higher BD-PRS was also significantly associated with reduced CTh of the left superior temporal gyrus (β = −0.32, *p* < 0.001) and right inferior temporal gyrus (β = −0.32, *p* = 0.049). Within the BD group (Fig. 2), higher BD-PRS was significantly associated with thinner left rostral middle frontal gyrus (β = −0.33, *p* = 0.03), left superior temporal gyrus (β = −0.33, *p* = 0.04), and right fusiform gyrus (β = −0.41, *p* = 0.01). Within the HC group (Fig. 3), higher BD-PRS was significantly associated with smaller CV of the bilateral superior frontal gyri (left: β = −0.46, *p* < 0.001; right; β = −0.38, *p* = 0.02) and smaller CSA of the left superior frontal gyrus (β = −0.45, *p* = 0.01). Correlations between significant clusters are presented in Fig. S2 in Supplementary Materials and Methods. Findings in the combined sample remained unchanged after controlling for diagnostic status. All findings remained significant after controlling for all 10 genetic principal components as well as BMI in sensitivity analyses. The findings on CTh also remained unchanged after controlling for ICV in sensitivity analyses.

**Table 2:** Association between BD-PRS and grey matter metrics of the significant clusters.

Cortical Metrics	MNI Coordinates			Cluster Size	cwp	β	Main Region	Additional Region(s)
	x	y	z					
**Significant clusters identified in combined sample of BD and HC**								
CV	−10.9	13.2	45.1	2996.86	0.002	−0.25	Left superior frontal gyrus	Caudal and rostral middle frontal gyri
CSA	−8.6	18.1	51.9	2961.54	0.03	−0.23	Left superior frontal gyrus	Caudal and rostral middle frontal gyri
CTh	−48.8	−20.4	−11.5	5381.29	<0.001	−0.32	Left superior temporal gyrus	Temporal pole; middle and inferior temporal gyri; fusiform gyrus
	42.6	−13.6	−25.9	1906.88	0.049	−0.32	Right inferior temporal gyrus	Fusiform and parahippocampal gyri
**Significant clusters identified within BD**								
CTh	−37.4	47	−1.4	2075.28	0.03	−0.33	Left rostral middle frontal gyrus	Caudal middle frontal gyrus; parstriangularis; lateral orbitofrontal cortex
	−51.1	7.9	−15.8	2021.08	0.04	−0.33	Left superior temporal gyrus	Middle and inferior temporal gyri
	41.4	−15	−24.2	2581.51	0.01	−0.41	Right fusiform gyrus	Parahippocampal gyri
**Significant clusters identified within HC**								
CV	−23	18.5	47.9	3985.42	<0.001	−0.46	Left superior frontal gyrus	Caudal and rostral middle frontal gyri
	21.8	0.8	51	2300.29	0.02	−0.38	Right superior frontal gyrus	Precentral, postcentral, and caudal middle frontal gyri
CSA	−23.3	16.5	47.4	3470.35	0.01	−0.45	Left superior frontal gyrus	Caudal and rostral middle frontal gyri

*MNI* Montreal Neurological Institute, *cwp* cluster-wise *p* value, *BD* bipolar disorder, *HC* healthy control, *CV* cortical volume, *CSV* cortical surface area, *CTh* cortical thickness.

In exploratory analyses there was no significant association of current or lifetime use of lithium, SGA, or lamotrigine with significant clusters identified within the BD group. However, there was a significant interaction between current and lifetime lithium use and BD-PRS on the right fusiform gyrus CTh cluster (current: β = 0.30, *p* = 0.02; lifetime: β = 0.31, *p* = 0.01), such that a significant negative association was found in BD participants without current or lifetime lithium use (current: β = −0.58, *p* < 0.001; lifetime: β = −0.59, *p* < 0.001), whereas no significant association between BD-PRS and right fusiform gyrus CTh was found in BD participants with current or lifetime lithium use (current: β = 0.62, *p* = 0.19; lifetime: β = 0.47, *p* = 0.27). Additionally, there was a significant interaction between BD-PRS and current SGA use on the right fusiform gyrus CTh cluster (β = −0.26, *p* = 0.04), such that a significant negative association was found in BD participants with current SGA use (β = −0.64, *p* < 0.001), while non-significant negative association was found in BD participants without current SGA use (β = −0.35, *p* = 0.21). No significant interaction between BD-PRS and lifetime SGA use and current or lifetime lamotrigine use was identified.

Female participants had significantly larger left superior frontal gyrus CV cluster (*p* = 0.04) and numerically smaller left rostral middle frontal gyrus CTh cluster (*p* = 0.05) than male participants, while no significant interaction between BD-PRS and sex were found on significant clusters identified from primary analyses. Sex-stratified associations between BD-PRS and grey matter metrics are presented in Figure S4 in Supplementary Materials and Methods.

### PRS and white matter integrity

**Fig. 4:**
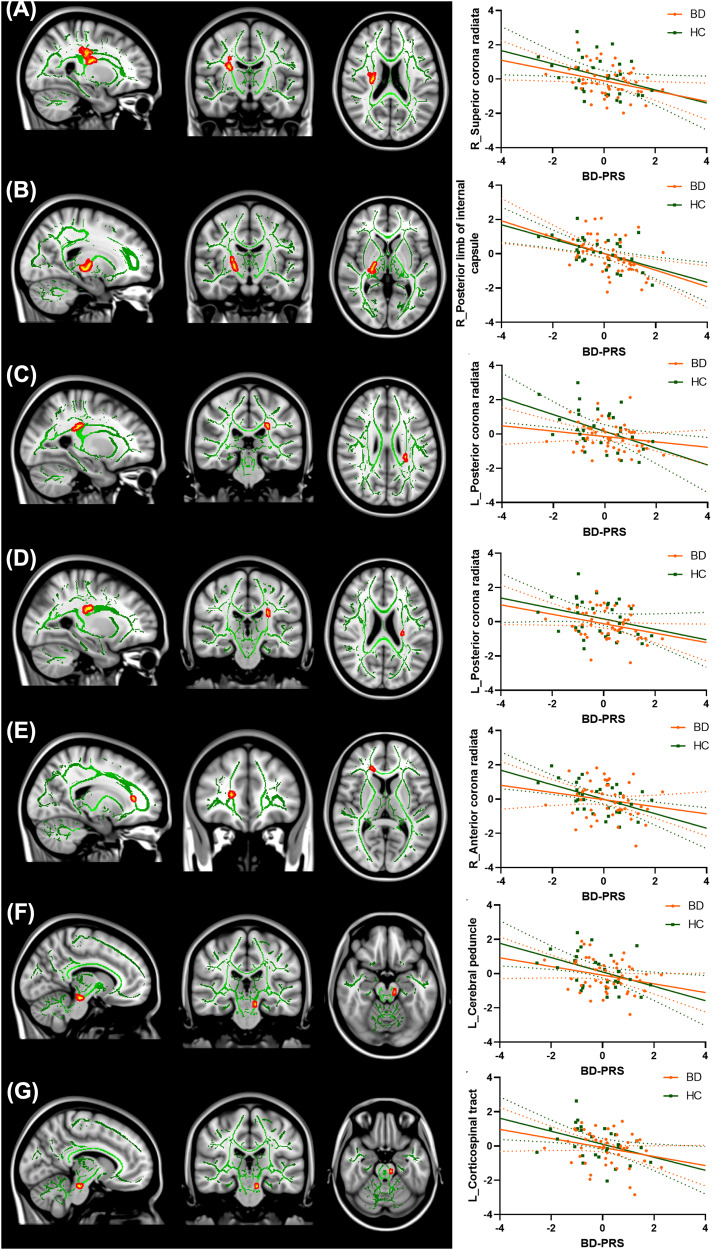
Association between BD-PRS and FA values of significant clusters identified in the combined sample of BD and HC. Higher BD-PRS was associated with lower FA in (**A**) right superior corona radiata, (**B**) right posterior limb of internal capsule, (**C**) left posterior corona radiata, (*D*) left posterior corona radiate, (**E**) right anterior corona radiata, (**F**) left cerebral peduncle, and (**G**) left corticospinal tract. The *y*-axis is labeled by the main region of significant clusters, and the value on *y*-axis indicates the standardized residuals of FA values of the significant clusters, adjusted for age, sex, and two genetic principal components.

In the combined sample, higher BD-PRS was associated with lower FA in 7 clusters (Table 3, Fig. 4), with peaks in: right superior corona radiata (cluster 1; β = −0.39, *p* = 0.04), right posterior limb of internal capsule (cluster 2; β = −0.47, *p* = 0.04), left posterior corona radiata (cluster 3, β = −0.36, *p* = 0.048; cluster 4, β = −0.35, *p* = 0.049), right anterior corona radiata (cluster 5, β = −0.33, *p* = 0.049), left cerebral peduncle (cluster 6, β = −0.37, *p* = 0.049), and left corticospinal tract (cluster 7, β = −0.36, *p* = 0.049). There were no significant associations between BD-PRS and FA from voxel-wise analyses within BD or within HC. Correlations between significant clusters are presented in Fig. S3 in Supplementary Materials and Methods. Findings in the combined sample remained unchanged after controlling for diagnostic status. Results remained significant after controlling for all 10 genetic principal components as well as BMI in sensitivity analyses.

**Table 3:** Association between BD-PRS and fractional anisotropy (FA) in the combined sample of BD and HC.

Cluster Number	MNI Coordinates			Voxel Numbers	*p*Max	β	Main region	Additional region(s)
	*x*	*y*	*z*					
1	64	113	92	377	0.04	−0.39	Right superior corona radiata	Posterior limb of internal capsule; Superior longitudinal fasciculus; Posterior corona radiata
2	70	112	74	329	0.04	−0.47	Right posterior limb of internal capsule	Cerebral peduncle; Inferior longitudinal fasciculus; Retrolenticular part of internal capsule;
3	115	96	99	70	0.048	−0.36	Left posterior corona radiata	Anterior thalamic radiation; Superior longitudinal fasciculus
4	116	103	94	46	0.049	−0.35	Left posterior corona radiata	Superior corona radiata; Anterior thalamic radiation
5	71	161	80	35	0.049	−0.33	Right anterior corona radiata	Forceps minor; Genu of corpus callosum
6	100	101	54	31	0.049	−0.37	Left cerebral peduncle	Anterior thalamic radiation; Corticospinal tract
7	99	101	49	25	0.049	−0.36	Left corticospinal tract	Anterior thalamic radiation

*MNI* Montreal Neurological Institute, *p*Max peak *p* value.

Exploratory analyses revealed that female participants had significantly smaller FA than male in left cerebral peduncle cluster (*p* = 0.04) and left corticospinal tract cluster (*p* = 0.01). Significant interaction between BD-PRS and sex on FA were found in the same clusters (β = 0.23, *p* = 0.04; β = 0.29, *p* = 0.01; respectively), where significant negative associations between BD-PRS and FA were found in male participants (β = −0.63, *p* = 0.002; β = −0.69, *p* = <0.001; respectively) but not in female participants (β = −0.13, *p* = 0.44; β = −0.11, *p* = 0.5; respectively). Sex-stratified associations between BD-PRS and FA are presented in Figure S5 in Supplementary Materials and Methods.

## Discussion

The present study adds to the limited literature regarding the associations of BD-PRS with grey matter structure and white matter integrity in youth, and extends prior findings by examining this topic in a youth BD sample. In the combined sample of BD and HC, there were significant negative associations between BD-PRS and superior frontal gyrus CV and CSA as well as superior and inferior temporal gyri CTh. In the within-group analyses, higher BD-PRS was associated with lower CTh of rostral middle frontal, superior temporal, and fusiform gyrus in BD, whereas higher BD-PRS was associated with lower CV and CSA of superior frontal gyrus in HC. Finally, in the combined sample, there were significant negative associations between BD-PRS and FA in various white matter regions, including superior corona radiata, posterior limb of internal capsule, posterior corona radiata, anterior corona radiata, cerebral peduncle, and corticospinal tract.

Our primary analyses in the combined sample of BD and HC found that higher BD-PRS was associated with smaller grey matter metrics in frontal and temporal regions. Specifically, higher BD-PRS was associated with smaller CV and CSA of the left superior frontal gyrus, which is involved in higher cognitive functions, particularly working memory [69]. Higher BD-PRS was also associated with thinner left superior temporal gyrus and right inferior temporal gyrus. The superior temporal gyrus has been implicated as a critical structure in social cognition [70], while inferior temporal gyrus plays a role in visual recognition and memory, and conversion from visual to reward information [71]. Grey matter reduction in these regions has been reported in both adults and youth with BD [18, 72–76] and may correspond to the observed cognitive impairments in working memory, social cognition, visual learning and memory, and reward processing [77–79]. Similar to the current findings, a prior study found that higher BD-PRS was associated with reduced CTh in a combined sample of adults with BD and HC[33], although another study reported no association between BD-PRS and subcortical volume in the combined sample [38].

Within the BD group, higher BD-PRS was significantly associated with thinner left rostral middle frontal gyrus, left superior temporal gyrus, and right fusiform gyrus, which have been previously implicated in BD [20, 73–76] and are involved in emotion regulation, working memory, social cognition, and face emotion perception [70, 80, 81]. A similar association has been observed in adults with BD, wherein higher BD-PRS was associated with thinner ventromedial prefrontal cortex [33]. Within the HC group, higher BD-PRS was significantly associated with smaller CV of the bilateral superior frontal gyri and smaller CSA of the left superior frontal gyrus. This is in line with the liability-threshold model of BD [82], as polygenic liability for BD is associated with neurostructural differences in youth even in the absence of BD. Similar negative associations between BD-PRS and grey matter volume have been observed in healthy adults [35], albeit in different regions, whereas mixed findings have also been reported in adult [34, 39].

In within-group analyses, higher BD-PRS was associated with lower regional CTh in BD but with lower regional CSA and CV in HC. It has been suggested that the genetic underpinnings of CTh and CSA are distinct [83, 84], and CV is thought to be more closely related to CSA than CTh [85]. Convergent with these findings, the largest neuroimaging study in BD (N = 6503) reported lower CTh but not CSA in BD participants as compared to HC [18], suggesting that particular relevance of CTh to the pathophysiology of BD. However, the fact that different structural phenotypes and different regions were identified in BD vs. HC should be interpreted tentatively for several reasons: 1) significant findings emerged for all three grey matter metrics in the combined sample even when controlling for diagnosis, 2) within-group analyses were comparatively less strongly powered, which may contribute to the lack of overlapping findings, and 3) contemporary BD-PRS is based on common SNPs (minor allele frequency >1%) and explains ~8% of variance in BD diagnosis. Future studies should consider integrating additional genetic factors (eg. rare, complex, sex-chromosome, and/or mitochondrial genetic variants) together with environmental factors, which could have interaction effects with BD-PRS on brain phenotypes and risk to BD. Such approaches may better elucidate the differential susceptibility to genetic factors in youth with BD vs. HC, as well as the resilience pathway in these healthy youth, who demonstrated neuroimaging changes associated with higher polygenic risk to BD but did not develop BD.

Findings regarding FA largely aligned with grey matter findings as higher BD-PRS was associated with lower FA in widespread regions in the combined sample. Significant clusters encompassed the right superior corona radiata, right posterior lime of internal capsule, left posterior corona radiata, right anterior corona radiata, left cerebral peduncle, and left corticospinal tract. These regions have been associated with voluntary emotional expression, cognition processing, and motor and sensory pathways [86–89], and lower FA in these regions has previously been reported in adults and youth with BD [25, 26, 28, 29, 31, 90, 91]. Prior studies of adults at familial risk for BD [41] did not find significant associations between BD-PRS and FA. However, these studies did not include individuals with BD, which may conceal differences in FA in relation to BD-PRS, whereas our combined sample was highly enriched for BD. The discrepancies may also reflect the developmental stage differences of our adolescent population compared to adult populations. Future longitudinal studies are warranted to examine neurodevelopmental differences in the effects of BD-PRS over time among individuals with and without BD. Finally, the current study did not find significant associations between BD-PRS and FA separately in the BD and HC groups, which may be due to the smaller sample size of individuals with DTI images.

Exploratory analyses did not reveal any significant main effect of medication use on significant clusters identified in the BD group. However, we found significant interaction between current and lifetime lithium use and BD-PRS on the right fusiform gyrus CTh, such that a significant negative association between BD-PRS and right fusiform CTh was found in BD participants without lifetime lithium use, whereas a non-significant positive association was found in BD participants with current or lifetime lithium use. Previous studies have reported larger grey matter structure associated with lithium use, and that lithium may exert neuroprotective effects that counteract pathological processes in the brain of individuals with BD [18, 92, 93]. Conversely, a significant interaction between current SGA use and BD-PRS was also found on the right fusiform gyrus CTh, where a significant negative association was found in BD participants who were currently taking SGA, while a non-significant negative association was found in BD participants not currently taking SGA. This is consistent with prior findings of smaller grey matter structure associated with current SGA use [18, 47]. We did not examine medication use on white matter integrity within the BD group as no significant association between BD-PRS and FA was found in within-group analyses. Future studies with larger sample size that are designed to examine the effect of medications on brain structure in youth BD and in relation to BD-PRS are needed to validate current findings. In addition, the current study did not record the duration of medication treatment or medication dosage, which could have crucial impact on the BD-PRS x brain associations and should also be considered in the future studies examining this topic.

Exploratory analyses also revealed significant main effects of sex on 3 clusters identified from primary analyses. Specifically, female participants had significantly larger left superior frontal gyrus CV, smaller left cerebral peduncle FA, and smaller left corticospinal tract FA than male participants. Significant interaction between BD-PRS and sex was also found on left cerebral peduncle FA and left corticospinal tract FA, such that a stronger negative association was found in male participants. Sex differences in grey matter and white matter structure have been extensively studies in both adults and youth [94–97], and sex-specific genetic-phenotypic associations has been emphasized [98]. However, sex differences in the association of BD-PRS with neuroimaging phenotypes has yet to be examined. Future studies with larger samples are warranted to examine sex differences in the effect of BD-PRS on brain structure in youth BD using formal tests of interaction.

It is noteworthy that the current findings do not converge with three prior studies based on general pediatric population samples (age 9–11 years old) [42–44]. Specifically, two publications based on the Generation R cohort study reported no significant association between BD-PRS and brain volume of 10 regions of interest (ROIs) and FA of 12 white matter tracts [42, 43]. Another study of the Adolescent Brain and Cognitive Development (ABCD) study cohort applied principal component analysis (PCA) on structural and diffusion-weighted imaging data and multivariate canonical correlation analysis. This study reported significant mode of covariation between BD-PRS and higher global CTh, smaller white matter volumes of the fornix and cingulum, larger medial occipital surface area and smaller surface area of lateral and medial temporal regions, although the multivariate model showed limited generalizability [44]. The discrepancy between current findings and these prior studies is likely influenced by methodologic differences including (1) different age group (13–20 in current study vs. 9–11 in these three studies) and therefore differential neurodevelopmental stage [99]; (2) BD-enriched sample in current study vs. general population sample; (3) different PRS calculation method (PRS-CS-auto vs. LDpred [43]/PRSice [42]/PRSice-2 + PCA [44]) and GWAS summary statistics (Mullins et al. 2021 vs. Sklar et al. 2011 [42, 43]/Stahl et al. 2019 [44]); (4) different imaging analysis approach (vertex- and voxel-wise based vs. ROI based [42, 43]/PCA [44]). These differences limited comparability among different studies. Future studies using data from large consortium (eg. ENIGMA or ABCD study) and applying the same standardized methodology are needed to demonstrate replicability in this field.

It is important to note that although the current sample size is relatively large for a single-site imaging study in the youth BD population, it is still small for PRS analyses. Therefore, the current study was not powered to detect small effect sizes, especially for the within-group analyses. The small sample size may also increase the chance of false positive findings and inflate effect sizes, which should be taken into consideration when interpreting current findings and when designing future studies [100]. A larger sample size would better elucidate whether the effect of BD-PRS on brain structure differs by factors such as diagnostic status, BD subtype, symptomatic status, sex, and/or treatment. In addition, due to the limited literature on neuroimaging correlates of BD-PRS in youth BD, and the preliminary nature of this study and small sample size, we opted to include findings from multiple imaging modalities in a single manuscript in order to inform future larger studies. We have corrected for multiple comparisons within each imaging modality (CV, CSA, CTh, FA), but not across different modalities, deferring such an approach to future adequately powered studies.

There are few additional limitations should be considered. First, FA values represent summary measures derived from diffusion tensor eigenvalues, and reduced FA is thought to reflect demyelination, reduction in axonal density, and/or a loss in fiber bundle coherence [101–103]. Future study examining other DTI metrics such as radial, axial, or mean diffusivity would be necessary to provide additional information regarding the specific type of white matter integrity deficits associated with BD-PRS. Second, aligning with prior neuroimaging studies in BD [18, 104], we controlled for intracranial volume for analyses examining CV and CSA (but not CTh) and did not control for other global structural measures (eg. overall CSA and CTh). Future larger neuroimaging studies may consider more comprehensive multivariate modeling for these global grey matter metrics [105]. Third, the cross-sectional design precludes the investigation of BD-PRS in relation to progressive brain changes over time. Because early-onset BD is characterized by a more symptomatic course of illness, which has been associated with smaller grey matter structure and lower FA, longitudinal studies are needed to parse the effects of BD-PRS and illness burden on brain structure over time [18, 25, 106]. Finally, because PRS is a combined index of many individual SNPs, it does not allow pinpointing for particular genes contributing to brain changes or the detailed biological mechanism underlying the observed associations.

Overall, the current study provides evidence that polygenic risk for BD, determined based on adult GWAS, is associated with smaller grey matter structure and lower FA in youth. We also found evidence of subtle differences in within-group analyses, which highlights the importance of future studies evaluating for BD-related differences in the associations of BD-PRS with neuroimaging phenotypes. These findings contribute to the sparse literature regarding the polygenic underpinnings of BD-associated grey and white matter anomalies in youth. Future longitudinal studies are warranted to examine whether BD-PRS contributes to changes in developmental trajectories in youth with BD vs. HC and its interaction with the course of illness and long-term medication use.

### Supplementary information

Supplementary Materials and Methods

## Data Availability

The datasets used and/or analyzed during the current study are available from the corresponding author on reasonable request. The data are not publicly available due to privacy or ethical restrictions.
