# Supplementary material for: Association of polygenic risk for bipolar disorder with grey matter structure and white matter integrity in youth
Source: Transl Psychiatry. 2023 Oct 18;13:322. doi: 10.1038/s41398-023-02607-y (PMC10584947; doi:10.1038/s41398-023-02607-y)
Supplement: Supplementary file 1 — Supplementary Materials and Methods [file 41398_2023_2607_MOESM1_ESM.docx]

**Supplementary materials**

*Collection of study data*

Study data were collected and managed using REDCap electronic data capture tools hosted at Sunnybrook Health Sciences Centre and later at the Centre for Addiction and Mental Health. REDCap (Research Electronic Data Capture) is a secure, web-based software platform designed to support data capture for research studies, providing 1) an intuitive interface for validated data capture; 2) audit trails for tracking data manipulation and export procedures; 3) automated export procedures for seamless data downloads to common statistical packages; and 4) procedures for data integration and interoperability with external sources.^1,2^

*Clinical methods*

Comorbid diagnoses and clinical characteristics (e.g., psychosis, psychotropic and psychosocial treatment history) were collected during the K-SADS-interview. Mood symptoms were assessed through the K-SADS Mania Rating Scale and the K-SADS Depression Rating Scale were used to assess mood symptoms.^3,4^ Age of BD onset was defined as the age at which the individual first experienced an episode of mania or hypomania according to DSM-IV, or when study criteria for BD-NOS were met. “Any Anxiety Disorders” included generalized anxiety disorder, separation anxiety disorder, agoraphobia, and anxiety disorder not otherwise specified. SUD included alcohol or drug abuse or dependence. Lifetime nicotine use was ascertained via the K-SADS-PL and was computed as a “yes” or “no” variable. K-SADS-PL post-traumatic stress disorder screening questions was used to obtain information regarding lifetime history of sexual and/or physical abuse. Socioeconomic status was calculated using the Hollingshead Four-Factor Index.^5^ The family psychiatric history of all first- and second-degree relatives was determined using the Family History Screen. Participants’ global functioning over the current period (past month), most severe past, and highest level in the past year were measured by the Children’s Global Assessment Scale (CGAS).^6^

*DNA extraction & genotyping*

DNA extraction was performed using a chemagic MSM I DNA extractor (Perkin-Elmer, Waltham, MA) as per manufacturer’s instructions. The extracted DNA was quantified using Qubit 2.0 Fluorometer (LifeTechnologies, Toronto, ON, Canada) and diluted to 50 ng/µL for use in downstream genotyping applications. Prior to analysis, all DNA dilutions were assayed using a custom TaqMan genotyping assay (LifeTechnologies, Toronto, ON, Canada) according to manufacturer’s directions for the amelogenin region. This assay was used as a quality control measure to identify any gross errors when manually preparing the dilutions and/or plating the samples (primer and probe sequences available upon request). DNA samples were genotyped on Infinium Global Screening Arrays (v3; Illumina) using iScan Array Scanner following manufacturer procedures (Illumina, Vancouver, BC, Canada). All genetic sample processing (DNA extraction and genotyping) was performed by the CAMH Biobank and Molecular Core Facility. Technicians were blinded to study groups

*Genetic quality control and imputation*

Quality control was performed on genome-wide data (summarized in Figure S1). Individuals participants were excluded based on the following criteria: (1) have same genotypes with another participant in the study, (2) have a first or second degree relative in the sample, (3) self-reported sex differed from that estimated from the microarray results that couldn’t be resolved, (4) abnormally high genome-wide heterozygosity (> 3 SD above or below sample mean), (5) individuals with genetically non-European ancestry (6) participants who were outliers on the top 10 ancestry informative principal components (> 6 SD above or below sample mean), and (7) individuals with more than 5% missing genotype data. SNPs were excluded based on the following criteria: (1) deviation from Hardy-Weinberg equilibrium (*p* < 1 x 10^-6^), (2) minor allele frequency below 1%, and (3) missing rate greater than 5%. Data were imputed using Minimac4 via the Michigan Imputation Server at CAMH with 1000 Genomes CEU as reference data. Imputed SNPs were excluded if they had minor allele frequencies of less than 5%, missing genotype rates of more than 1%, genotypes that significantly deviated from Hardy-Weinberg Equilibrium (p<10^-6^), or poor imputation quality (imputation information score less than 0.7).

**
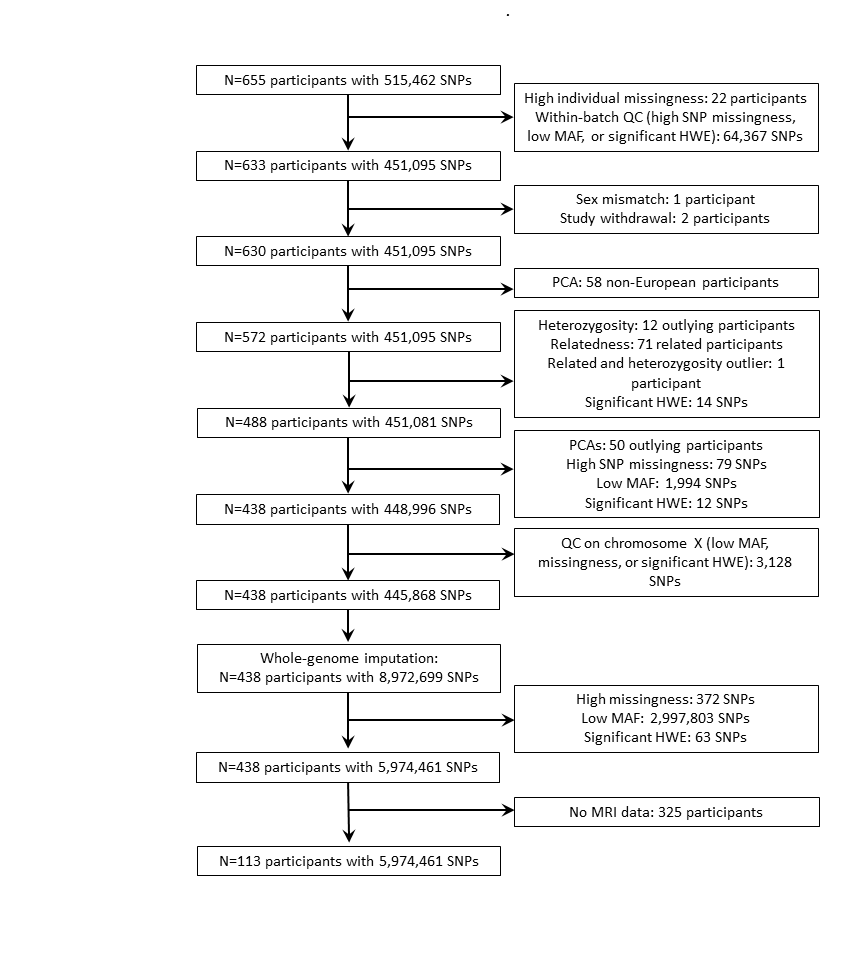
**

**Figure S1. Flowchart showing quality control (QC) steps for genome-wide genotype data and inclusion/exclusion of research participants in the study.** SNP, single nucleotide polymorphism; HWE, Hardy-Weinberg equilibrium; PCA, principal component analysis; MAF, minor allele frequency.

**
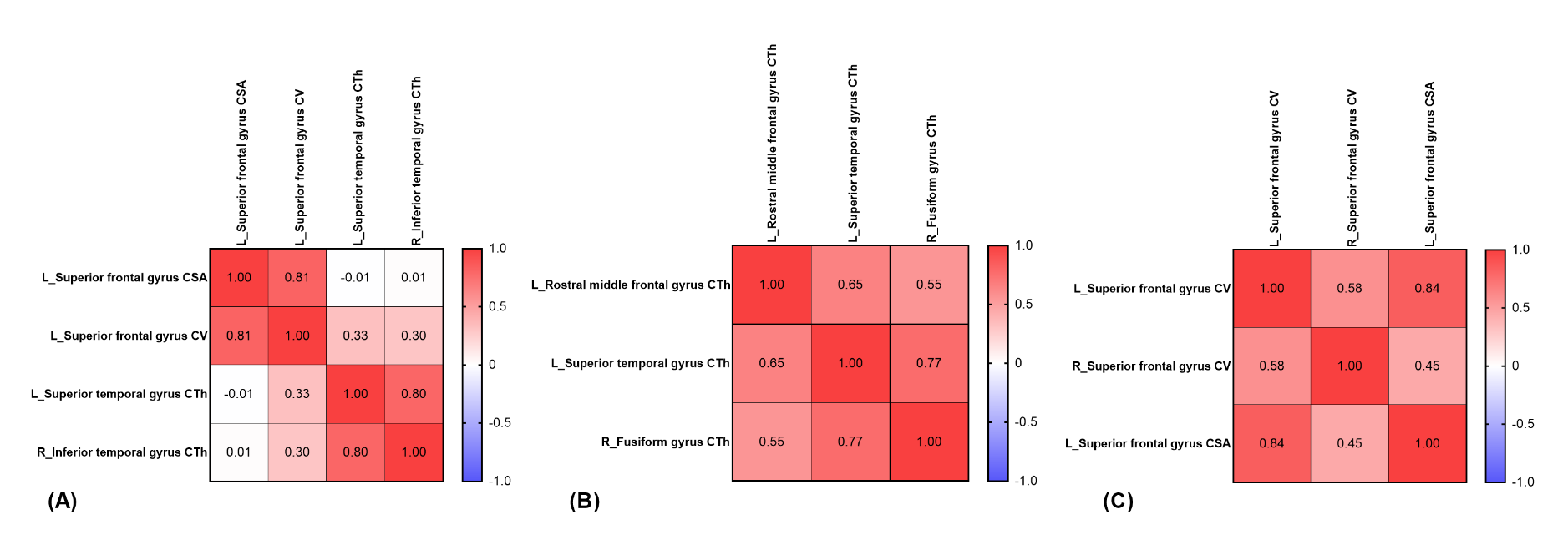
**

**Figure S2. Pearson correlations between grey matter metrics of the significant clusters identified in the (A) combined sample, (B) BD group, and (C) HC group.** All grey matter metrics were adjusted for age and sex. CV and CSA were also adjusted for intracranial volume. BD=bipolar disorder; HC=healthy control; CV=cortical volume; CSA=cortical thickness; CTh=cortical thickness.

**
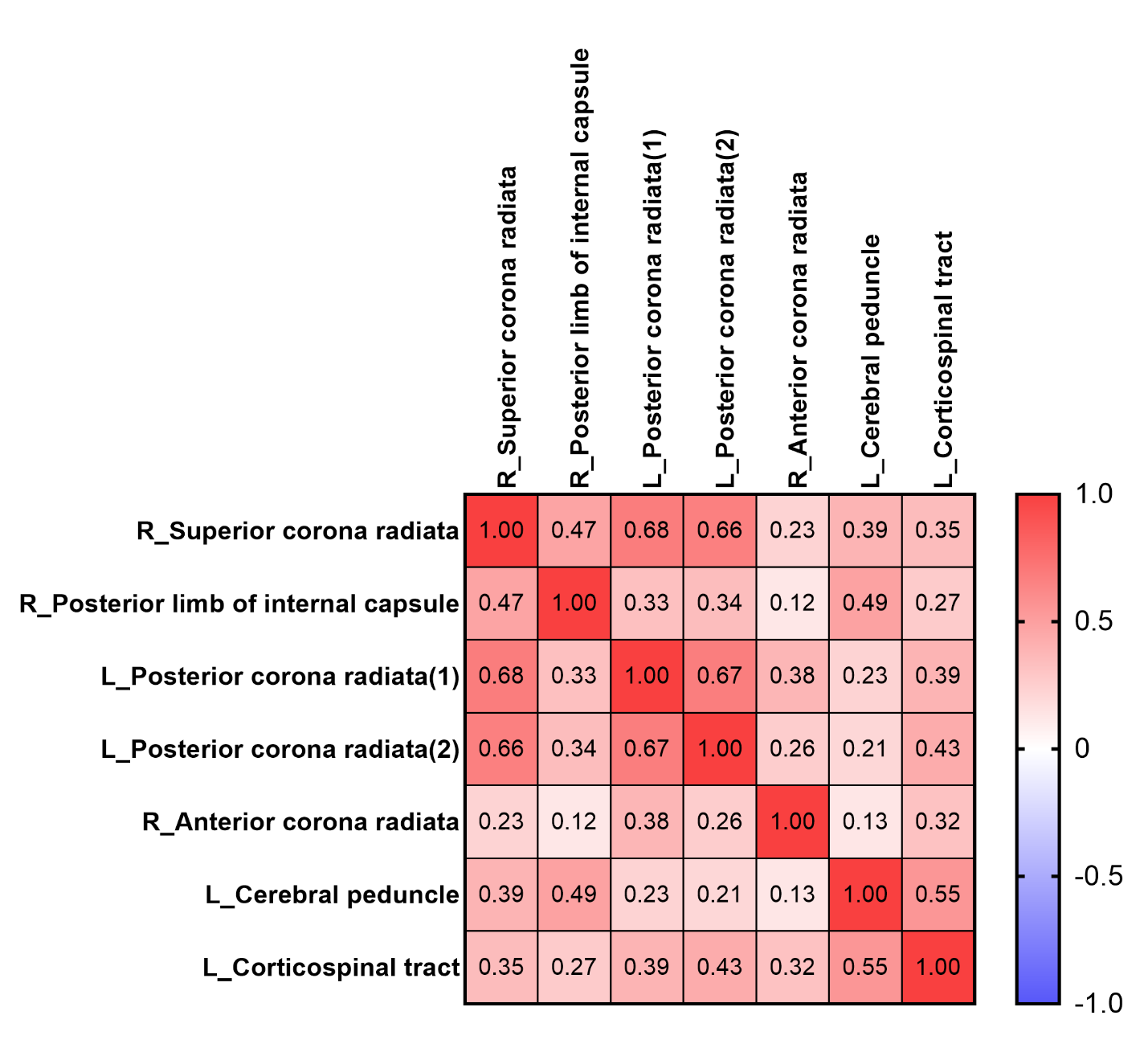
**

**Figure S3. Pearson correlations between FA values of significant clusters identified in the combined sample,** **adjusted for age and sex.**

**
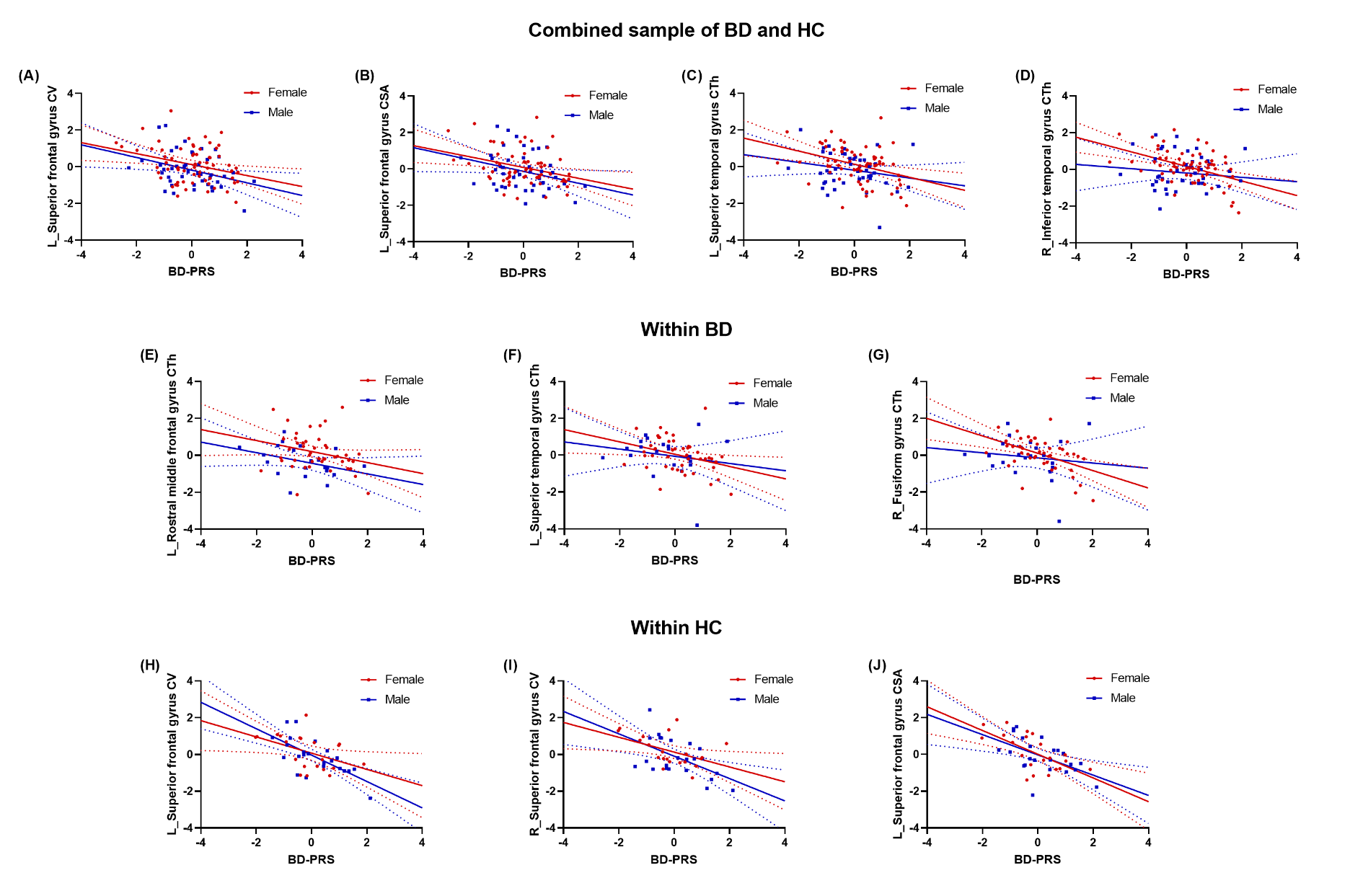
**

**Figure S4. Sex-stratified associations between BD-PRS and grey matter structure.** The y-axis is labeled by the main region of significant clusters, and the value on y-axis indicates the standardized residuals of grey matter metrics of the significant cluster, adjusted for age, sex, and two genetic principal components. CV and CSA were also adjusted for intracranial volume. CV=cortical volume, CSA=cortical surface area; CTh=cortical thickness.

**
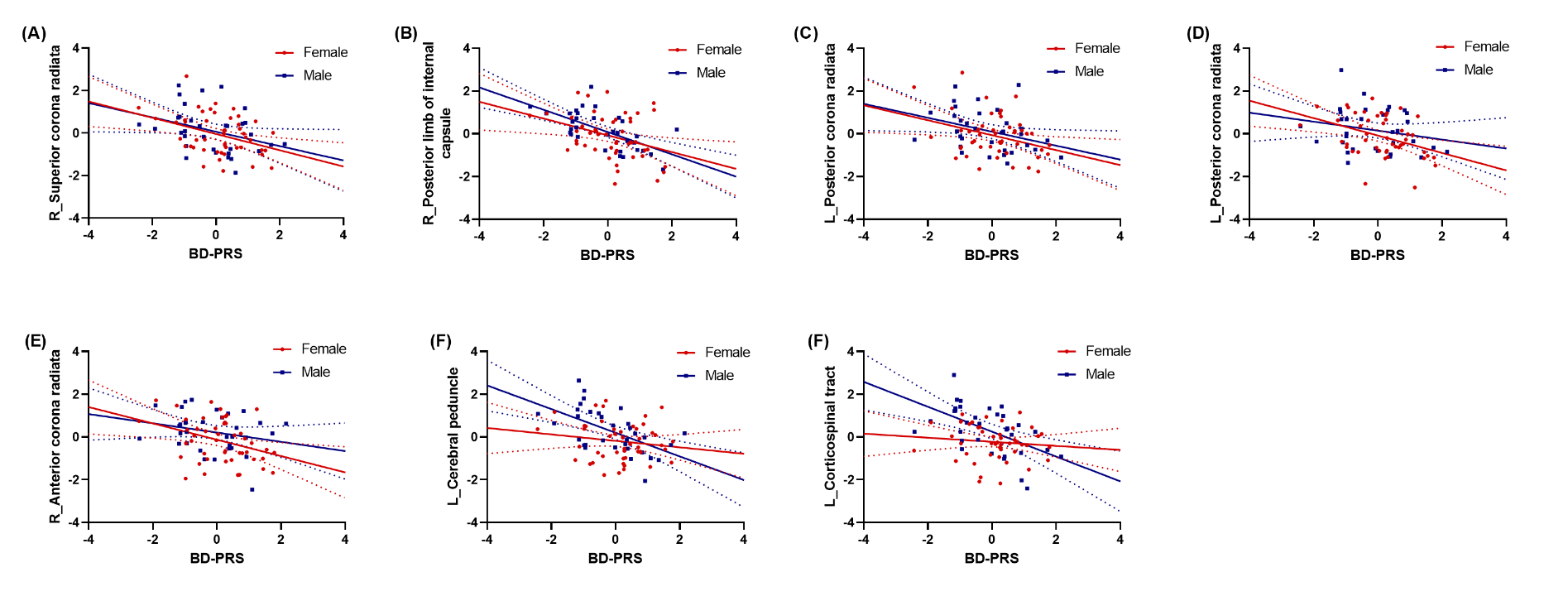
**

**Figure S5. Sex-stratified associations between BD-PRS and white matter integrity in the combined sample.** The y-axis is labeled by the main region of significant clusters, and the value on y-axis indicates the standardized residuals of FA values of the significant cluster, adjusted for age, sex, and two genetic principal components.

**Table S1. Clinical Characteristics of BD participants**

|  | **With T1-weighted images**  **(n=66)** | **With DTI images**  **(n=49)** |
| --- | --- | --- |
| BD-I | 21 (31.8%) | 18 (36.7%) |
| BD-II | 21 (31.8%) | 15 (30.6%) |
| BD-NOS | 24 (36.4%) | 16 (32.7%) |
| Age of BD Onset | 14.5±2.7 | 14.6±2.8 |
| Lifetime Psychosis | 16 (24.2%) | 13 (26.5%) |
| Lifetime Suicide Attempts | 11 (16.7%) | 7 (14.3%) |
| Lifetime Self-injurious Behaviour | 40 (60.5%) | 29 (59.2%) |
| Lifetime Suicidal Ideation | 50 (75.8%) | 37 (75.5%) |
| Police Contact/Arrest | 14 (21.2%) | 10 (20.4%) |
| Lifetime Physical Abuse | 2 (3.0%) | 2 (4.1%) |
| Lifetime Sexual Abuse | 3 (4.5%) | 3 (6.1%) |
| Lifetime Psychiatric Hospitalization | 29 (43.9%) | 20 (40.8%) |
| Current Depression Score | 15.4±11.2 | 16.1±11.1 |
| Lifetime Depression Score | 32.6±10.5 | 32.7±9.3 |
| Current Mania Score | 10.7±10.0 | 7.9±9.5 |
| Lifetime Mania Score | 30.6±11.2 | 31.8±10.4 |
| CGAS – Most severe past episode | 44.2±8.6 | 45.3±9.5 |
| CGAS – Highest past year | 67.4±10.6 | 71.4±8.7 |
| CGAS – Past month | 65.5±11.0 | 67.6±10.4 |
| **Lifetime Comorbid Diagnoses** |  |  |
| ADHD | 30 (45.5%) | 24 (49.0%) |
| Any Anxiety | 56 (84.8%) | 43 (87.8%) |
| SUD | 12 (18.2%) | 9 (18.4%) |
| ODD | 20 (30.3%) | 17 (34.7%) |
| CD | 3 (4.5%) | 2 (4.1%) |
| Nicotine Use (yes/no) | 31 (47.0%) | 21 (42.9%) |
| Alcohol Abuse | 5 (7.6%) | 4 (8.2%) |
| Alcohol Dependence | 4 (6.1%) | 2 (4.1%) |
| **Family Psychiatric History** |  |  |
| Mania/hypomania | 40 (60.6%) | 30 (61.2%) |
| Depression | 52 (78.8%) | 41 (83.7%) |
| Psychosis | 16 (24.2%) | 11 (22.4%) |
| ADHD | 23 (34.8%) | 19 (38.8%) |
| Anxiety | 47 (71.2%) | 37 (75.5%) |
| **Lifetime Medications** |  |  |
| SGA | 49 (74.2%) | 36 (73.5%) |
| Lithium | 14 (21.2%) | 12 (24.5%) |
| SSRI Antidepressants | 25 (37.9%) | 21 (42.9%) |
| Non-SSRI Antidepressants | 13 (19.7%) | 8 (16.3%) |
| Stimulants | 14 (21.2%) | 15 (30.6%) |
| Valproate | 4 (6.1%) | 2 (4.1%) |
| Lamotrigine | 18 (27.3%) | 13 (26.5%) |
| Any medications | 54 (81.8%) | 43 (87.8%) |
| **Current Medications** |  |  |
| SGA | 40 (60.6%) | 28 (57.1%) |
| Lithium | 12 (18.2%) | 11 (22.4%) |
| SSRI Antidepressants | 7 (10.6%) | 9 (18.4%) |
| Non-SSRI antidepressants | 5 (7.6%) | 2 (4.1%) |
| Stimulants | 5 (7.6%) | 7 (14.3%) |
| Valproate | 0 | 0 |
| Lamotrigine | 15 (22.7%) | 11 (22.4%) |
| **Note**. Values for all continuous variables are presented as mean ± standard deviation and categorical variables are presented as n (% within group).  BD=bipolar disorder; HC=healthy controls; NOS=not otherwise specified; Depression Score Based on Depression Rating Scale; Mania Score Based on Mania Rating Scale; CGAS=Children’s Global Assessment Scale; ADHD=attention-deficit/hyperactivity disorder; SUD=substance use disorder; ODD=oppositional defiant disorder; CD=conduct disorder; SGA=second generation antipsychotic; SSRI=selective serotonin reuptake inhibitor. | | |

**Table S2. Pearson correlations between intracranial volume and significant cortical thickness clusters.**

| **Cluster main region** | ***r*** | ***p*** |
| --- | --- | --- |
| *Cortical thickness clusters identified from analyses in the combined sample* | | |
| Left superior temporal gyrus | -0.03 | 0.79 |
| Right inferior temporal gyrus | -0.16 | 0.09 |
| *Cortical thickness clusters identified from analyses in the BD group* | | |
| Left rostral middle frontal gyrus | -0.02 | 0.91 |
| Left superior temporal gyrus | 0.07 | 0.59 |
| Right fusiform gyrus | -0.08 | 0.53 |

**Supplementary References**

1 Harris PA, Taylor R, Minor BL, Elliott V, Fernandez M, O’Neal L *et al.* The REDCap consortium: Building an international community of software platform partners. *J Biomed Inform* 2019; **95**: 103208.

2 Harris PA, Taylor R, Thielke R, Payne J, Gonzalez N, Conde JG. Research electronic data capture (REDCap)--a metadata-driven methodology and workflow process for providing translational research informatics support. *J Biomed Inform* 2009; **42**: 377–381.

3 Chambers WJ, Puig-Antich J, Hirsch M, Paez P, Ambrosini PJ, Tabrizi MA *et al.* The assessment of affective disorders in children and adolescents by semistructured interview. Test-retest reliability of the schedule for affective disorders and schizophrenia for school-age children, present episode version. *Arch Gen Psychiatry* 1985; **42**: 696–702.

4 Axelson D, Birmaher BJ, Brent D, Wassick S, Hoover C, Bridge J *et al.* A preliminary study of the Kiddie Schedule for Affective Disorders and Schizophrenia for School-Age Children mania rating scale for children and adolescents. *J Child Adolesc Psychopharmacol* 2003; **13**: 463–470.

5 Hollingshead AB. Four factor index of social status. 1975.https://sociology.yale.edu/sites/default/files/files/yjs_fall_2011.pdf#page=21.

6 Shaffer D, Gould MS, Brasic J, Ambrosini P, Fisher P, Bird H *et al.* A children’s global assessment scale (CGAS). *Arch Gen Psychiatry* 1983; **40**: 1228–1231.
